# Diagnosis of Myelodysplastic Syndromes: From Immunological Observations to Clinical Applications

**DOI:** 10.3390/diagnostics12071659

**Published:** 2022-07-07

**Authors:** Yannick Simoni, Nicolas Chapuis

**Affiliations:** 1 Institut Cochin, Université Paris Cité, CNRS UMR8104, INSERM U1016, 75014 Paris, France; yannick.simoni@inserm.fr; 2 Assistance Publique-Hôpitaux de Paris, Centre-Université Paris Cité, Service d’Hématologie Biologique, Hôpital Cochin, 75014 Paris, France

**Keywords:** myelodysplastic syndromes, clonal hematopoiesis of indeterminate potential, inflammation, aging, innate immunity, adaptive immunity

## Abstract

Myelodysplastic syndromes (MDS) constitute a very heterogeneous group of diseases with a high prevalence in elderly patients and a propensity for progression to acute myeloid leukemia. The complexity of these hematopoietic malignancies is revealed by the multiple recurrent somatic mutations involved in MDS pathogenesis and the paradoxical common phenotype observed in these patients characterized by ineffective hematopoiesis and cytopenia. In the context of population aging, the incidence of MDS will strongly increase in the future. Thus, precise diagnosis and evaluation of the progression risk of these diseases are imperative to adapt the treatment. Dysregulations of both innate and adaptive immune systems are frequently detected in MDS patients, and their critical role in MDS pathogenesis is now commonly accepted. However, different immune dysregulations and/or dysfunctions can be dynamically observed during the course of the disease. Monitoring the immune system therefore represents a new attractive tool for a more precise characterization of MDS at diagnosis and for identifying patients who may benefit from immunotherapy. We review here the current knowledge of the critical role of immune dysfunctions in both MDS and MDS precursor conditions and discuss the opportunities offered by the detection of these dysregulations for patient stratification.

## 1. Introduction

Myelodysplastic syndromes (MDS) represent a heterogeneous group of clonal or oligoclonal disorders affecting hematopoietic stem and progenitor cells (HSPCs), with a high prevalence in elderly patients. These diseases are characterized by ineffective hematopoiesis, peripheral cytopenia, dysplastic morphology, genetic instability and a propensity for transformation to acute myeloid leukemia (AML) [1,2]. In most cases, MDS are preceded by an asymptomatic phase with preserved blood counts termed clonal hematopoiesis of indeterminate potential (CHIP) [3], during which abnormal hematopoietic stem cells (HSCs) harboring mutations found in hematological malignancies can be detected in the bone marrow (BM) [4]. Other precursor states of MDS are now also well defined, such as clonal cytopenia of undetermined significance (CCUS) and idiopathic cytopenia of undetermined significance (ICUS), both characterized by relevant cytopenia without morphologic dysplasia but, respectively, with or without somatic mutation confirmed by high-throughput sequencing (HTS) [5]. At MDS diagnosis, the revised International Prognostic Scoring System (IPSS-R) by encompassing different parameters such as the depth of cytopenia, BM blast percentage and cytogenetic subsets allows the identification of different groups of patients with a high/very high, intermediate or low/very low risk of progression to AML and will therefore guide treatment decisions tailored to each case [6]. However, the IPSS-R is not considered as an efficient tool for predicting response to treatment such as erythropoiesis-stimulating agents (ESAs) or hypomethylating agents (HMAs). Furthermore, relative rapid progression of MDS can still occur in some MDS patients classified as low risk according to the IPSS-R. Recent advances in HTS have led to the identification of a dynamic genomic landscape with multiple recurrent somatic mutations involved in MDS pathogenesis [7,8,9]. Although not disease-defining as they are not fully specific to MDS, accumulating evidence suggests that the identification of recurrent mutations and thus clonal hematopoiesis is highly informative for predicting outcome and treatment response [10,11], and for increasing therapeutic options with novel molecular targets. Considering that MDS display marked heterogeneity regarding prognosis and risk of disease progression to AML, the monitoring of each patient at diagnosis in daily clinical practice remains challenging. Furthermore, given the global aging of the population, the incidence of MDS and their precursor states (CHIP, CCUS, ICUS) will strongly increase in the next decades. A better understanding of the process leading to the progression of CHIP to MDS and finally to AML therefore becomes timely and imperative. 

It is also well known that a dynamic interplay between mutated hematopoietic cells and both mesenchymal and immune cell populations is critical to the pathogenesis and the outcome of MDS. Indeed, it was recently suggested that aberrant innate immune signaling and a pro-inflammatory environment could have a central role in the pathogenesis of MDS and could contribute to explain, at least partially, the paradoxical common hematological phenotype observed despite the high molecular complexity of MDS [12]. Clinical and laboratory findings clearly suggested that immune dysregulations may represent a key driver for the genesis and/or evolution of MDS, but their characterization is not integrated into the diagnosis workflow of MDS. However, different dysregulations and dysfunctions of the immune system can be dynamically observed during the course of the disease. Monitoring the immune system at the time of MDS diagnosis could therefore be very helpful to more precisely assess the stage of the disease and the inherent risk of progression to AML for each case of MDS, in addition to current prognosis models based on cytogenetic and mutational data. Finally, deep characterization of deregulated immune pathways could also offer perspectives for the use of immunomodulatory drugs in some subtypes of MDS specifically adapted to the patient’s immune status as already recently reviewed [13], and this will probably lead to the development of new targeted therapies. Here, we therefore review the different immune dysregulations which can be detected during the course of MDS, i.e., from the MDS precursor conditions (CHIP, CCUS, ICUS) to their leukemic progression. We also discuss how these data could be integrated for routine diagnosis and risk stratification of these malignant or premalignant neoplasms and their clinical implications for treatment decisions. 

## 2. Innate Immune Signaling in Response to Inflamm-Aging in the Context of CHIP

The incidence of MDS markedly increases in elderly people, making advanced age the greatest risk factor for developing MDS [14]. It is well known that during the process of aging, the hematopoietic system is characterized by (1) an increased number of myeloid-biased hematopoietic stem cells (HSCs), (2) a decreased number of B-cell progenitors and (3) an expansion of memory B and T cells [15]. Altogether, this leads to a spontaneously increased level of pro-inflammatory cytokines/chemokines and a functional decline of the immune system [16]. Although it cannot be excluded that this hematopoietic and immune remodeling represents an adaptive phenomenon necessary for human longevity [17], it is currently accepted that these processes of immune senescence and inflamm-aging result in infections, cancers, autoimmune disorders and chronic inflammatory diseases in the elderly. Furthermore, aging is frequently associated (10–20% of individuals with age >70) with clonal expansion of specific HSCs carrying recurrent somatic mutations including DNMT3a, TET2 and ASXL1 [18]. The presence of clonal hematopoiesis attested by a variant allele frequency (VAF) of >2% of a somatic mutation in a hematologic malignancy-associated gene in a subset of individuals without a known hematologic cancer or other clonal state defines the precursor condition of myeloid neoplasm termed CHIP. Indeed, the presence of CHIP was associated with a ~10-fold increased relative risk of these malignancies over several years of follow-up [3,19]. In addition to the increased risk of hematological malignancies, CHIP is also associated with a 30–40% increased mortality risk [19,20]. However, this risk is reported to be due rather to atherosclerotic cardiovascular disease and ischemic stroke [21], and an increased incidence of pulmonary disease [22,23] and type 2 diabetes [24,25]. 

Interestingly, there is emerging evidence that these mutations are implicated in the regulation of innate immunity. This was suggested by the frequent detection of mutations associated with CHIP with a similar allelic burden in CD34+ progenitors, granulocytes and monocytes, but also in lymphoid cells such as natural killer (NK) cells. These mutations were also detected in T and B cells with a lower VAF [26], but differences were observed according to the type of CHIP mutation [26,27]. Indeed, in patients carrying the DNMT3a CHIP mutation, T cells were mutated in 30 to 50% of cases but rarely in patients with TET2, ASXL1 or SF3B1 mutations [26]. The involvement of CHIP-associated genes in lymphoid cell function is also highlighted by the detection of TET2 and DNMT3a mutations in human T-cell lymphoma [28,29]. The higher rates of graft-versus-host disease and the reduced relapse rates observed in patients who received a BM transplantation from a donor carrying a CHIP mutation also reveal the probable role of these genes in the regulation of the adaptive immune response [30,31]. 

**Figure 1:**
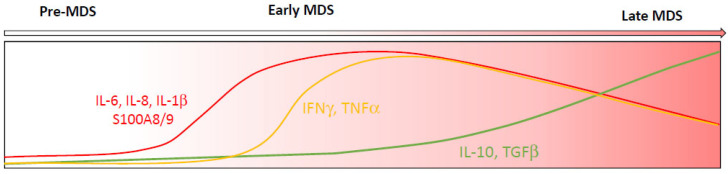
Schematic diagram showing a relative timescale for cytokines produced during MDS progression. The pro-inflammatory cytokines IL-6, IL-8, IL-1β and S100A8/9 are detected first during pre/early MDS (red), followed by the pro-inflammatory cytokines IFNγ and TNFα (yellow). Suppressive cytokines IL-10 and TGFβ (green) start to be detected during early MDS and increase during MDS progression.

Recent studies have also precisely described the role of TET2 and DNMT3a in immunity. Mice deficient in Tet2 developed more severe inflammation in response to endotoxin or low-density lipoprotein stimulation attested by the up-regulation of several inflammatory mediators, including IL-6, and the increased expression of Il1b and IL-8 family chemokines in Tet2-deficient macrophages [18,32,33,34]. Similarly, Dnmt3a inactivation promoted the expression of Cxcl1, Cxcl2, IL-6 and Ccl5 in an LPS-stimulated macrophage cell line [35]. In mast cells lacking Dnmt3a, an exacerbated response to IgE stimulation characterized by a higher level of IL-6, TNF-α and IL-13 was also observed [36]. Several studies also reported a high level of pro-inflammatory cytokines such as IL-6 and IL-8 in individuals with CHIP [21,37]. Accordingly, a similar pro-inflammatory cytokine profile including TNF-α, INF-γ, TGF-β, IL-6 and IL-8 has also been observed in patients with low-risk MDS [38,39]. The global level of pro-inflammatory and suppressive cytokines from pre-MDS conditions to the late phase of MDS is shown in Figure 1.

## 3. Selective Expansion of MDS Clones at the Early Phase of MDS Is Driven by Innate Immune Dysregulation

Aging and either MDS or pre-MDS HSPCs themselves clearly concomitantly contribute to the creation of a chronic inflammatory BM environment which promotes the expansion of mutated HSPCs over normal HSPCs and therefore the risk of MDS development. However, the mechanisms of how preleukemic or MDS HSPCs selectively acquire a clonal dominance over normal HSPCs are not precisely understood. Evidence recently emerged to highlight the role of the aberrant innate immune activation within the malignant clone and the BM microenvironment in the competitive advantage of MDS HSPCs over normal HSPCs during low-grade and chronic inflammation. 

The innate immune system is known to be activated following the detection of pathogen-associated molecular patterns (PAMPs) or host cell-derived danger-associated molecular patterns (DAMPs) by pattern recognition receptors (PRRs) such as Toll-like receptors (TLRs) or cytosolic nucleotide-binding domain and leucine-rich repeat pattern recognition receptors (NLRs) [40]. Activation of these PRRs promotes the recruitment of intracellular adaptors (TIRAPS, MyD88), kinases (IRAK, TRAF6) and effector molecules to form a complex called an inflammasome. This platform serves to activate caspase 1 through pyrin-domain containing receptors (NLRPs) which can lead to the inflammatory-mediated cell death pyroptosis. HSCs express innate immune receptors and are therefore able to directly respond to inflammatory mediators [41]. In the context of aging, chronic activation of the TLR signaling due to prolonged inflammation impaired normal hematopoiesis through activation of the NLRP3 inflammasome, leading to the functional decline of HSCs [42,43]. Recent reviews precisely described the different innate immune dysregulations on HSCs from patients with either MDS or their precursor states [12,44]. The expression of immune-related genes in HSPCs was reported to be frequently increased in MDS patients [45]. TLRs were reported to be overexpressed on HSCs and progenitor cells of MDS patients compared with controls but with specific patterns along the course of the disease, therefore suggesting that TLR expression likely influences the pathogenesis of MDS in lower-risk patients [46,47,48,49,50]. An increased level of the DAMP S100A8/9, an endogenous ligand of TLR4, and downstream activation of the NLRP3 inflammasome are also well described in MDS mainly in low-risk patients [51,52,53,54,55]. S100A8/A9, through the activation of NADPH oxidase (NOX), induces increased generation of reactive oxygen species (ROS), resulting in inflammasome assembly and stabilization of β-catenin [52]. This signaling pathway leads to pyroptosis of MDS cells and therefore contributes to the common feature observed in MDS patients i.e., ineffective hematopoiesis resulting in cytopenia(s) [56]. Accordingly, this NLRP3–pyroptosis axis was found to be activated in primary MDS samples regardless of the mutation profile detected by HTS [52]. In contrast, interestingly, the pyroptosis level can be monitored by quantification through flow cytometry of plasmatic ASC specks, adaptor apoptosis-associated speck-like proteins containing a caspase recruitment domain that accumulate following inflammasome assembly, to provide an index of medullary pyroptotic cell death and ineffective hematopoiesis in patients with MDS [57]. In this study, the authors showed that the peripheral blood (PB) ASC speck percentage was significantly higher in MDS patients compared to control samples, especially in low-risk MDS. Notably, the percentage of ASC specks also increased with the number of mutations detected by HTS, attesting, therefore, the relevance of this parameter in the physiopathology of these diseases and its usefulness for detecting specific MDS-associated inflammation, especially in low-risk MDS patients. The pyroptosis-mediated cell death also induces the release of active IL-1β and IL-18 and other intracellular proteins that contribute to local inflammation, and maintains this inflammatory circuit [52]. Furthermore, S100A8/A9 stimulates the expansion of myeloid-derived suppressor cells (MDSCs) in the BM of patients with MDS which, in turn, also secrete S100A8/A9, therefore leading to an amplification of inflammatory signals [58]. However, in this context of increased inflammatory-mediated cell death, the expansion of the mutated MDS clone of cells remains paradoxical. Interestingly, recent evidence has emerged to understand the mechanisms that give a competitive advantage to MDS HSCs in this inflammatory environment. Indeed, activation of NLRP3 by S100A8/A9 drives β-catenin activation, a signaling pathway known to promote leukemia stem cell self-renewal [52]. Furthermore, in both mouse models of MDS and primary samples, Muto et al. observed that MDS HSPCs exhibited a preferential activation of the noncanonical NF-κB pathway which protects these cells from chronic inflammation, while normal HSPCs exhibited activation of canonical NF-κB signaling [59]. The switch from canonical to noncanonical NF-κB signaling is dependent on the TLR-TRAF6-mediated activation of A20, a dual ubiquitin editing enzyme that suppresses TRAF6-mediated canonical NF-κB activation while simultaneously having the ability to induce noncanonical NF-kB activation through the stabilization of NIK. Interestingly, the competitive advantage of TLR-TRAF6-primed HSPCs could be restored by deletion of A20 or inhibition of the noncanonical NF-κB pathway, therefore suggesting that interfering with noncanonical NF-κB signaling could prevent MDS progression [59].

Although the NLRP3–pyroptosis axis was found to be activated regardless of the mutation profile detected by HTS [52], some innate immune deregulations were found to be specifically associated with some molecular and/or cytogenetic abnormalities. For example, many investigations clearly demonstrated that genetic alterations such as the deletion 5q (del(5q)) directly lead to abnormal activation of innate immunity. Indeed, del(5q) induces miR-145 and miR-146a haploinsufficiency, therefore resulting in inappropriate activation of innate immune signaling in MDS HSCs due to overexpression of (1) Toll–interleukin-1 receptor domain–containing adaptor protein (TIRAP), which is important to Myddosome formation, and (2) tumor necrosis factor receptor–associated factor-6 (TRAF6), a protein adaptor that lies in the TLR-MyD88 pathway [60,61]. In mice, knockdown of miR-145 and miR-146a together or enforced expression of TRAF6 in mouse HSPCs phenocopies several clinical features of del(5q) MDS (thrombocytosis, mild neutropenia and megakaryocytic dysplasia) and can lead to marrow failure or AML [61]. Notably, a typical del(5q) phenotype with severe anemia and pathognomonic megakaryocyte morphology was observed in mice with combined deficiency of different genes within the deleted segment in del(5q) MDS such as Rps14/Csnk1a1/miR-145/146a [62]. Furthermore, deletion in mice of the DIAPH1 gene located on 5q31.3, which encodes mDia1, also leads to cytopenia and myeloid dysplasia characterized by increased TLR4-IL6 signaling and a specific up-regulation of CD14 messenger RNA in granulocytes [63,64,65]. Mechanistically, the severe anemia observed in patients with del(5q) MDS was also demonstrated to be linked to the abnormal innate immune signaling induced by the p53-dependent increase in the endogenous TLR ligand S100A8/A9 [66]. Indeed, genetic inactivation of S100a8 expression rescues the erythroid differentiation defect of Rps14-haploinsufficient HSCs, whereas simple addition of recombinant S100A8 is sufficient to induce a differentiation defect in wild-type erythroid cells [66]. Similarly, in another model characterized by del(20q), a frequent cytogenetic abnormality detected in hematologic malignancies such as MDS but also in myeloproliferative neoplasm (MPN), the STK4 gene encoding for the Hippo kinase MST1 was identified as a key 20q deleted gene with a central role in the biology of del(20q)-associated hematologic malignancies. Indeed, loss of MST1 induces hyperactivation of innate immune signaling through IRAK1 and downstream activation of the NF-κB pathway that may be therapeutically exploitable via IRAK1 inhibition [67]. Finally, a direct link between a specific MDS-associated mutation and innate immune deregulation was shown in the context of U2AF1 mutation. Indeed, mutation in the splicing factor U2AF1 leads to the production and the overexpression in MDS HSPCs of a longer hyperactive isoform of Interleukin-1 receptor associated kinase 4 (IRAK4-L). In contrast to the normal isoform expressed in normal HSPCs, this longer isoform can directly bind MyD88 for assembling the Myddosome, therefore leading to the maximal activation of NF-κB even in the absence of receptor activation with ligands which can be therapeutically targeted [68]. However, the expression of the hyperactive isoform IRAK4-L is also detected in MDS with SF3B1 mutation or without an underlying splicing factor mutation, hence the common implication of enhanced innate immune activation in MDS pathogenesis [68,69].

## 4. Clinical Immune Manifestation in MDS

From a clinical point of view, systemic inflammatory and autoimmune diseases (SIAD) are frequently observed in MDS patients, with the incidence ranging from 10 to 28% in the overall MDS population [70,71,72,73,74]. This increased incidence in MDS is confirmed by the lower prevalence of SIAD in the general population or other neoplastic entities such as lymphoma or breast cancer [75,76,77]. Clinical manifestations are very heterogeneous but systemic vasculitis, connective tissue diseases and hypothyroidism were reported to be the most frequent immune phenomena associated with MDS [70,71]. Interestingly, the onset of SIAD frequently precedes the diagnosis of MDS (31–37% of cases) but can also be detected concomitantly (7–31%) or during the course of MDS (32–61%), with a mean of 8.6 months between the diagnosis of the two diseases [71,74]. The strong association between the two diseases is attested by the increased risk of developing myeloid malignancies such as MDS and/or AML in patients with different SIAD [78,79]. Furthermore, a hematologic improvement is observed in nearly half of the selected patients with MDS receiving immunosuppressive treatment such as ciclosporin or antithymocyte globulin [80,81,82,83]. Finally, the interconnection between MDS and autoimmunity is reinforced by the fact that, in most MDS patients treated with HMA, SIAD response was achieved in 80–86% of cases [71,72]. Interestingly, this high sensitivity to hypomethylating agents could be linked to the specific pattern of mutation more frequently observed in MDS patients with SIAD (TET2/IDH in 59% vs. 38% of patients with or without SIAD, respectively) and the better response to HMA observed in patients with a TET2 mutation [10]. Furthermore, the authors of this study also revealed a specific immune pattern in patients with TET2/IDH mutations characterized by a reduction in Treg, naive/memory T-cell imbalance and defects in immune checkpoint receptor expression. Their results suggest a more activated status of T cells with a reduced control against autoimmunity which could explain the higher incidence of SIAD in these MDS patients. Somatic mutation in the gene encoding for UBA1 has been recently described in patients with adult-onset inflammatory diseases, defining a novel disease called VEXAS (vacuoles, E1 enzyme, X-linked, autoinflammatory, somatic) syndrome which is frequently associated with MDS (25 to 50% of VEXAS cases) [84,85]. The presence of the UBA1 mutation was detected in some MDS patients with SIAD (12%) [86]. However, in contrast to other MDS patients with SIAD, those harboring a UBA1 mutation tend to be less sensitive to HMA [86]. 

## 5. Abnormal Immune Cell Repartition and/or Functions during the Course of MDS

**Figure 2:**
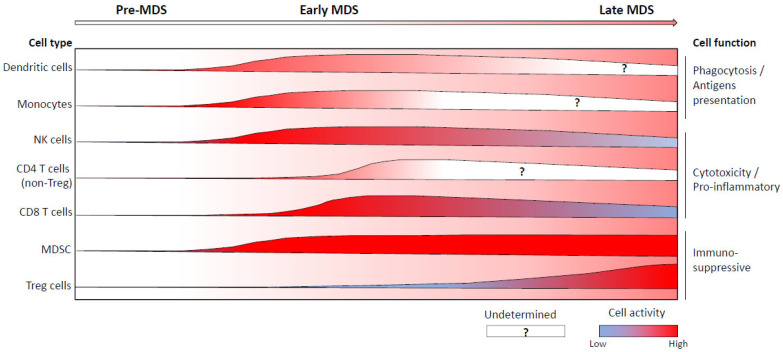
Amount and intrinsic activity of the key immune cell subsets implicated in the genesis and/or the evolution of MDS. The thickness of the curve represents the number of cells detected during MDS progression. For each curve, the color gradient represents the activation status for functions described on the right of the figure (blue, low activity; red, high activity; white, absence of information about cell activity).

The repartition and the function of a large number of immune cell subsets have been investigated in MDS patients. The global amount and activity of these cells are shown in Figure 2. 

### 5.1. Natural Killer Cells

Among these immune populations, many studies specifically focused on cytotoxic T cells and NK cells which are well known to be critical in host defense against malignant transformation and to be potent anti-leukemic cytotoxic effectors. Initially, it was shown that MDS patients have a severe and selective deficiency of IFN-gamma-producing natural killer T (NKT) cells [87]. Furthermore, although the peripheral NK cell population was quantitatively normal in most MDS patients [88], a small subgroup of patients have a selective deficiency in these cells which is associated with a poor prognosis [89]. Moreover, a phenotypic shift from the mature to the immature state was observed in NK cells from MDS patients and was shown to constitute an adverse factor for overall survival [89,90,91]. In addition, NK cells from MDS patients exerted a highly decreased constitutive cytolytic activity partially due to down-regulation of the activating receptors NKp30 and NKG2D or reduced levels of perforin and granzyme B [88,89,92]. The altered NK function was also significantly associated with a higher IPSS, an abnormal karyotype, excess blasts and BM hypercellularity. Altogether, these data clearly suggest, therefore, that the inefficient generation of mature, functionally competent natural killer cells observed in MDS probably contributes to MDS disease progression through impaired immune surveillance [92], and that restoring the full anti-tumor potential of these cells could be an attractive therapeutic strategy for these patients as previously reviewed [93]. Thus, monitoring the quantity of NK cells, their maturation and their cytotoxic function through the expression of killer-cell immunoglobulin-like (KIR) receptors represents an opportunity to assess the global immune state of the patient at MDS diagnosis and to potentially evaluate the progression risk independently of the IPSS-R. Furthermore, it could also be helpful for predicting response to treatment such as HMA. Indeed, it was shown in vitro that HMA treatment enhanced NK cell-mediated recognition of malignant cells by increasing the expression of KIR [94]. However, a basal severe impairment of NK cytotoxicity was shown to be associated with a shorter duration of response to 5-azacytidine and shorter survival [95].

### 5.2. T Lymphoid Cells

T cells play a central role in immune responses against tumor cells. They express a T-cell receptor (TCR) that can specifically recognize tumor antigens. Based on their functional properties, different populations have been identified: (1) CD8^+^ cytotoxic T cells act directly by killing tumor cells; (2) CD4^+^ helper T cells organize the immune response against tumor cells; and (3) T regulatory cells (Treg) suppress the immune response. A clear understanding of the role of each subset in MDS pathology, according to disease severity and treatment, represents an opportunity for the development of new therapies targeting these populations (i.e., immunotherapy).

### 5.3. CD8^+^ Cytotoxic T Cells

After recognition of tumor antigens presented by tumor cells through their MHC class I, activated CD8^+^ T cells release cytotoxic molecules leading to tumor cell apoptosis [96]. In the BM of MDS patients, a decrease in CD8^+^ T-cell cytotoxicity has been observed [97,98]. This loss of cytotoxicity is confirmed by the observation of an exhausted-like phenotype of bone marrow CD8^+^ T cells in MDS. This phenotype is characterized by an up-regulation of inhibitory receptors such as PD-1 [99], Tim-3 [100,101] or TIGIT [102]. Studies have shown that the exhaustion and loss of cytotoxicity on T cells are defining features of many cancers [103]. T-cell dysfunction is influenced by the microenvironment, notably the cancer cell and immunosuppressive cell populations (see below the MDSC and Tregs sections). This exhausted phenotype can be reverted by the use of immune checkpoint blocking antibodies, such as anti-PD-1 therapy (i.e., nivolumab, pembrolizumab). Although preclinical studies suggested potential benefits of anti-PD-1 treatment, clinical trial outcomes after applying this treatment as a monotherapy are disappointing [104]. However, when combined with HMA, treatment with anti-PD-1 shows improvement in clinical response [104].

Clonal CD8^+^ T-cell expansion is observed in the BM of low-risk MDS patients [105]. This expansion is associated with a skewed TCR repertoire, suggesting a clonal expansion of tumor-specific CD8^+^ T cells [105,106]. The identification of tumor antigens represents a promising approach for the development of immunotherapies in MDS, such as tumor antigen vaccination or tumor-specific T-cell adoptive transfer. The tumor antigen Wilms’ tumor gene 1 (WT1) is expressed in MDS patients [107]. The presence of WT1-specific CD8^+^ T cells is observed in the BM of MDS patients [108]. Phase I clinical trials of vaccination with WT1 are safe and well tolerated and demonstrate the induction of a tumor-specific CD8^+^ T-cell response in MDS patients [109,110]. Similarly, vaccination of MDS patients with the NY-ESO-1 tumor antigen induces a tumor-specific CD8^+^ T-cell response [111]. Among tumor antigens, neoantigens (i.e., antigens derived from mutations specific to tumor cells) represent a target of interest. Phase I clinical trials demonstrate that neoantigen-specific CD8^+^ T cells from MDS patients can be expanded in vitro and are capable of killing autologous tumor cells [112]. Infusion of these neoantigen-specific CD8^+^ T cells into MDS patients is safe and well tolerated [109]. Overall, these data demonstrate that MDS patients could be good candidates for immunotherapies targeting CD8^+^ T cells, but treatment efficacy could be improved by the identification of appropriate therapeutic targets (e.g., tumor antigens shared by a group of patients) and a better delineation of the patient subgroups. Additional studies are needed to assess the dysfunctional status of these cells according to MDS stages.

### 5.4. CD4^+^ Helper T Cells

After recognition of tumor antigens presented by antigen-presenting cells through their MHC class II, activated CD4^+^ helper T cells (Th cells) release cytokines which are essential for antigen-presenting cells and CD8^+^ T-cell activation. Accumulating evidence has shown the ability of CD4^+^ T cells to eradicate tumor cells [113]. According to their cytokine production profile, CD4^+^ helper T cells are divided into distinct subsets: Th1 (IFN-γ), Th2 (IL-4), Th17 (IL-17), Th22 (IL-22). Th1 cells are detected in the BM of MDS patients, with a decreased frequency compared to control individuals [114]. No abnormalities in Th2 cell response have been reported. The frequency and the function of Th17 cells were elevated in low-risk MDS while being decreased in high-risk MDS [115]. This observation was closely related to clinical parameters including karyotype and blast percentage [116]. In late-stage MDS patients, an increased frequency of Th22 cells is observed in their blood, suggesting a role for these cells in this group of patients [116]. Furthermore, an up-regulation of the inhibitory receptor PD-1 (up to 50% of Th cells) is observed in the BM of MDS patients [99]. These observations highlight an abnormal profile of Th subsets in MDS. Additional studies are needed to assess these abnormal Th cell profiles according to phenotypic markers (e.g., CTLA-4, TIGIT) and correlated with MDS stages. Furthermore, tumor antigen-activating Th cells remain to be identified to confirm the role of CD4^+^ helper T cells in MDS pathology.

### 5.5. Regulatory T Cells

Many studies also focused their attention on the possible involvement of regulatory T cells (Tregs) in MDS pathogenesis. Indeed, given their central role in maintaining immune tolerance, these cells are known to be expanded in malignant diseases, therefore contributing to the suppression of host anti-tumor responses. In MDS, an initial study observed a significant correlation between the increased number of CD4+ Tregs in the blood and MDS subgroups according to the percentage of blast cells, the IPSS and the disease progression, making the quantification of CD4+ Tregs a potential biomarker of high-risk MDS [117]. Indeed, the accumulation and activity of Tregs seem to follow the course of the disease, as the early phase of MDS is characterized by impaired BM homing of Tregs due to down-regulation of CXCR4, whereas the function and the migratory capacity are maintained in late-stage MDS, therefore allowing their BM expansion [118,119]. Interestingly, in patients with low-risk MDS, an altered Treg compartment was detected in 34.6% of cases [120]. Notably, a unique Treg subset (effector memory Tregs) characterized by greater suppressive activity in vitro was increased in some of these patients and negatively impacted the survival independently from myeloblast characteristics, cytopenia, karyotype and comorbidities [120]. Another study clearly demonstrated the prognostic value of the level of Tregs in the blood for the survival of patients with lower-risk MDS [121]. More recently, expansion of highly immunosuppressive Tregs characterized by an ICOS^high^/PD-1^neg^ phenotype was shown to be a highly significant independent predictor of inferior overall survival and more specifically associated with TP53 mutation [122]. The global expansion of Tregs, or more specifically of a more immunosuppressive subpopulation of Tregs, therefore marks a pivotal point of immune escape in MDS and constitutes a primary driver of poor outcomes in these diseases [123]. Accordingly, it was also observed that a decreased number of Tregs in the BM was associated with the expansion of cytotoxic T lymphocytes in low-risk MDS patients, therefore suggesting that the frequency of these cells at diagnosis inversely associates with an immune profile able to control disease progression [105]. Similar to NK cells, treatment with HMA seems to impact the functions of Tregs as the accumulation of Tregs and both their proliferative capacity and suppressive functions can be reduced by HMA [124,125]. 

### 5.6. Myeloid-Derived Suppressor Cells 

More recently, different studies demonstrated the involvement of myeloid-derived suppressor cells (MDSCs) in the global immunosuppression which is observed during the course of MDS. Indeed, these cells are known to accumulate in patients with cancer and to contribute to tumor progression through their suppressive activity toward T cells partially driven by inflammation-associated signaling molecules, such as the DAMP heterodimer S100A8/S100A9 [126]. The first clear evidence of the implication of MDSCs in MDS pathogenesis came from the study of Chen et al. [58] which demonstrated their accumulation in the BM of MDS patients and their critical role in the development of ineffective hematopoiesis. Indeed, their expansion is driven by the interaction of the pro-inflammatory molecule S100A9 with CD33, which then stimulates the secretion by MDSCs of suppressive cytokines such as IL-10 and TGF-β. Although the identification of MDSCs remains challenging especially in MDS due to the very frequent abnormal expression of different myeloid markers on both granulocytes and monocytes [127], the increased number of MDSCs in blood samples from MDS patients was confirmed in other studies [128,129,130]. Similar to other immune cell populations, the accumulation of MDSCs seems to follow the course of the disease as their number is lower in very low/low-risk patients compared to intermediate/high/very high risk patients and correlates with Treg quantification [128]. Furthermore, MDSCs in MDS patients express a specific pattern of BM-homing chemokine receptors (CXCR4, CX3CR1) which probably contributes to their accumulation in the BM. Furthermore, in addition to their accumulation, the immunosuppressive function of MDSCs was suggested to be increased in higher-risk MDS [131]. The suppression of CD8^+^ T lymphocyte function was also shown to be mediated by different mechanisms such as the STA3-ARG1 or TIM3/Gal-9 pathways [101,129]. Altogether, these findings clearly identify a critical role of MDSCs in the dysregulation of immune surveillance in MDS and lead, therefore, to the development of therapeutic strategies directly targeting MDSC functions in MDS patients [132,133,134,135]. 

### 5.7. Mesenchymal Stem Cells

Given their key role in supporting hematopoiesis and their immune modulatory properties, mesenchymal stem cells (MSCs) have been extensively evaluated in MDS. In low-risk MDS patients, an impaired immuno-modulatory function was first described [136]. Significant differences in the immunoregulatory functions between low-risk and high-risk MDS MSCs were also detected. Compared to low-risk MDS MSCs, high-risk MDS MSCs are characterized by an increased TGF-β1 expression and a higher immunosuppressive rate, partially due to a stronger inducible rate of Tregs. Moreover, MSCs from MDS patients can acquire phenotypic and metabolic properties of myeloid-derived suppressor cells (MDSCs), with resulting suppression of NK cell function, along with T-cell proliferation [112]. Furthermore, in a murine model of Shwachman–Diamond syndrome (SDS), a rare congenital disorder notably characterized by ineffective blood cell production, MSCs were shown to induce, through an abnormal p53-mediated secretion of S100A8/A9, mitochondrial hyperpolarization and an increase in ROS and DNA damage response. Interestingly, the capacity of MSC-derived S100A8/A9 to induce genotoxic stress was also confirmed in human HSPCs [137]. In addition, MSCs also induced suppressive monocytes with reduced expression of the TGF-β transcriptional repressor MAB21L2 and with increased INHBA expression, a gene that encodes for a member of the TGF-β superfamily of proteins [112]. Thus, these results indicate the different immunoregulatory roles of MSCs in MDS compared to healthy controls, but also according to their progression risk, which may be important for understanding the pathogenesis of MDS and the development of novel immunomodulatory strategies for the treatment of these diseases. 

### 5.8. Monocytes and Macrophages

Concerning the cells of the monocytic/macrophage lineage, it has been shown that the degree of BM apoptosis and the level of inflammatory cytokines correlate with the number of monocytes/macrophages in patients’ BM [138]. Furthermore, BM monocytes from MDS patients displayed up-regulation of TLR-4 and its downstream signaling pathway, which contributed to the production of inflammatory cytokines [56]. In this study, it was also demonstrated that patients’ macrophages had an impaired capacity to engulf apoptotic cells, which maintains and/or enhances an inflammatory BM microenvironment through an excessive release of high mobility group box-1 protein (HMGB1) by dying cells [56]. Although the number of monocytes is increased in MDS patients [139,140], their ability to differentiate into macrophages is reduced [139]. The phagocytosis activity of monocytes from MDS patients was also shown to be altered, with a low expression of genes involved in triggering immune responses, regulating immune and inflammatory response signaling pathways, and the response to LPS [139,141]. Furthermore, dendritic cells derived in vitro from MDS monocytes also demonstrated a qualitatively and quantitatively altered cytokine secretion [142] and a reduced expression of HLA-DR and CD86, suggesting that antigen-processing and T-cell activation capabilities are impaired [141]. This probably contributes to the increased susceptibility to infections observed in MDS patients. Recently, the number of PB monocyte subsets according to CD14 and CD16 expression was shown to be a key biomarker for the diagnosis of chronic myelomonocytic leukemia (CMML) [143,144]. Interestingly, accumulation of classical monocytes (CD14+ CD16-) was also detected in some MDS patients, which frequently evolved into CMML [145]. Another recent study revealed abnormal quantitative and functional characteristics of monocyte subsets in lower-risk MDS, substantiating their role in the immune deregulation associated with the disease. Indeed, MDS patients had a significantly increased number of intermediate monocytes with an increased ability to produce TNFα following stimulation with LPS, and differential gene expression mostly associated with biological pathways/processes relevant to hematopoiesis, immune signaling and cell adhesion [146].

## 6. Perspective on Immune Monitoring at MDS Diagnosis

**Figure 3:**
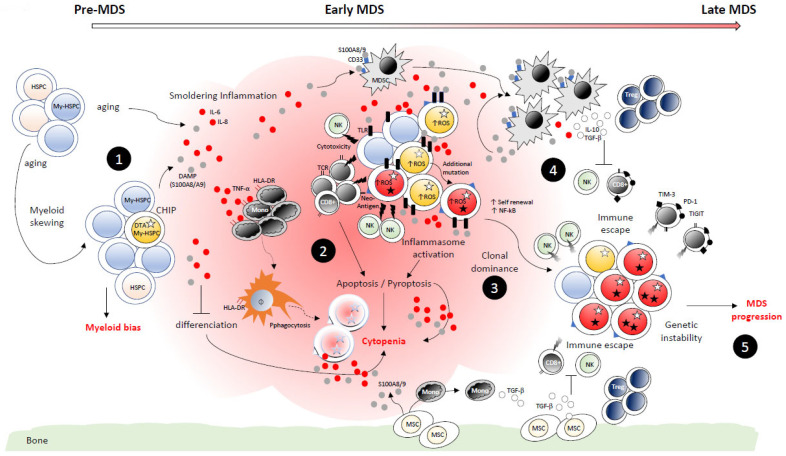
Immune dysregulations during the course of MDS. (**1**) With aging, the hematopoietic system is characterized by a myeloid bias and smoldering inflammation. In addition, aging is frequently associated with clonal expansion of specific HSPCs carrying recurrent somatic mutations including DNMT3a, TET2 and ASXL1 (DTA). Altogether, this leads to increased pro-inflammatory levels of cytokines (IL-6, IL-8) and DAMPs such as S100A8/A9. (**2**) Chronic inflammation through activation of the inflammasome induces the death of both normal and MDS HSPCs. Furthermore, CD8^+^ T cells associated with NK cells exhibit their cytotoxic activity toward both normal and MDS HSPCs, leading to the apoptosis of these cells. DAMPs such as S100A8/A9 also impair hematopoietic cell differentiation. Altogether, this leads to ineffective hematopoiesis and cytopenia, the common phenotype observed in early-phase MDS. In addition, the decreased number and phagocytic activity of macrophages allow the release by dying cells of pro-inflammatory cytokines and DAMPs. Monocytes and MSCs also release pro-inflammatory cytokines such as TNF-α and S100A8/A9, respectively. This therefore contributes to the maintenance and amplification of pro-inflammatory signals and induces deeper cytopenia. (**3**) In the context of increased inflammatory-mediated cell death, the expansion of the mutated MDS clone remains paradoxical. However, increased self-renewal and intrinsic activation of alternative signaling pathways protect these cells from chronic inflammation and finally promote the expansion of MDS HSPCs. (**4**) DAMPs such as S100A8/A9 induce MDSC expansion through interaction with CD33. These cells, in turn, release S100A8/A9 to enhance their own expansion but also immunosuppressive cytokines (IL-10 or TGF-β). Recruitment of highly immunosuppressive Tregs in the BM is also concomitantly increased, therefore leading to inhibition of both CD8^+^ T cells’ and NK cells’ cytotoxicity. In addition, the intrinsic impaired cytotoxicity of NK cells and the up-regulation of inhibitory receptors’ expression (TIM-3, PD-1 and TIGIT), leading to T-cell exhaustion, induce a global immune evasion which enhances the clonal dominance of MDS HSCs. This immune escape is also promoted by MSCs which directly (or through monocyte reprogramming) release immunosuppressive cytokines (TGF-β). (**5**) The global immune evasion promotes the expansion of MDS clones and the progression of the disease through genetic instability.

All the immune dysregulations reviewed herein and shown in Figure 3 clearly support the role of immune monitoring at diagnosis as an important additional and independent factor for the stratification of patients with MDS, and more specifically low-risk patients according to the IPSS-R. Indeed, identification of patients with either a signature of smoldering inflammation or an immunogenic or immunosuppressive profile could potentially more precisely indicate the stage of the disease and guide treatment decisions notably for the use of immunotherapies. An Immunoscore, based on the analysis of immune cells that infiltrate the cancer and surround it [147], is now fully integrated into the standardized diagnosis of solid cancers such as colorectal and colon cancers for predicting both survival and response to chemotherapy [148,149,150]. Identification of immune cells such as T and B cells, monocytes and dendritic cells by multiparametric flow cytometry (MFC) is also recommended by the European LeukemiaNet International MDS-Flow Cytometry Working Group in patients with suspected MDS [151]. However, until now, there is no consensus about the method for global analysis of immunity in hematopoietic malignancies including MDS. Perspectives on the integration of the “Immunome” in the stratification of MDS were recently discussed with a proposed highly attractive strategy based on the combination of multi-omics-driven analysis with either HTS, single-cell RNAseq, CITE-seq, mass cytometry (CyTOF) or imaging mass cytometry [152]. Other innovative molecular techniques such as the use of liquid biopsies to detect alterations in nucleic acids in peripheral blood by HTS or digital PCR are also promising and could be useful in the future for the diagnosis, prognosis and response to treatment of hematological malignancies including MDS, as recently reviewed [153]. However, among these technologies, only HTS is currently available at MDS diagnosis in daily clinical practice. Interestingly, MFC is now a well-recognized tool that contributes to the diagnosis of MDS and also to the prognostication and the prediction/evaluation of response to therapy of MDS patients [151]. Currently, MFC analysis is mainly focused on the characterization of myeloid progenitor cells and maturation pathways of granulocytes, monocytes or erythroid cells in the BM [127,154,155,156,157]. Given the increased number of parameters already detected with routine flow cytometers currently and the attractive perspectives concerning the transfer to the clinic in the future of new technologies such as spectral flow or CyTOF, exploration of immune cell populations by MFC represents, therefore, a clearly feasible strategy which could be applied in routine daily practice. Questions about the precise panels of markers which have to be used preferentially and the types of immune cell populations which have to be analyzed remain to be answered. That is why prospective studies based on the combination of clinical data with multi-omics immunophenotypic data obtained with CyTOF or spectral flow technologies are clearly needed in order to have, in paired BM and PB MDS samples, a large view of the immune landscape in MDS patients at diagnosis and during the follow-up of the disease with or without treatment. CyTOF analyses have already been performed in MDS samples, but they were more specifically focused on myeloid populations [158,159]. Deciphering the immune profiles of MDS patients with such strategies will therefore allow the identification of the most relevant markers we have to use and the cell populations we have to focus on for routine analysis.

## 7. Conclusions

In conclusion, it is now clearly accepted that immune dysregulations and dysfunctions have, in combination with specific molecular defects, a critical role in MDS pathogenesis and contribute to the clinical evolution of these diseases, from MDS pre-conditions to leukemic progression. From a clinical point of view, immune dysregulation can be clinically relevant as SIAD are frequently observed in MDS patients and could lead, in these MDS cases, to the use of immunosuppressive therapies. Monitoring the innate and adaptive immune systems in MDS patients clearly requires the deep analysis of immune cell population repartition and function and/or the detection of an inflammatory or immunosuppressive microenvironment by cytokine profile analysis. Integration of data reflecting immune deregulation into the diagnosis workflow of MDS could be very informative for more precisely predicting the progression risk to leukemia and identifying patients who may benefit from novel therapeutic strategies such as vaccination therapies or direct targeting of inflammasome activation. Multiparametric studies on large cohorts of MDS patients are therefore necessary in order to identify key immune biomarkers and immune cell populations which have to be carefully analyzed for routine clinical investigations at MDS diagnosis.

## References

[1] Cazzola M. (2020). Myelodysplastic Syndromes. N. Engl. J. Med..

[2] Adès L., Itzykson R., Fenaux P. (2014). Myelodysplastic Syndromes. Lancet Lond. Engl..

[3] Jaiswal S., Ebert B.L. (2019). Clonal Hematopoiesis in Human Aging and Disease. Science.

[4] Sperling A.S., Gibson C.J., Ebert B.L. (2017). The Genetics of Myelodysplastic Syndrome: From Clonal Haematopoiesis to Secondary Leukaemia. Nat. Rev. Cancer.

[5] DeZern A.E., Malcovati L., Ebert B.L. (2019). CHIP, CCUS, and Other Acronyms: Definition, Implications, and Impact on Practice. Am. Soc. Clin. Oncol. Educ. Book.

[6] Greenberg P.L., Tuechler H., Schanz J., Sanz G., Garcia-Manero G., Solé F., Bennett J.M., Bowen D., Fenaux P., Dreyfus F. (2012). Revised International Prognostic Scoring System for Myelodysplastic Syndromes. Blood.

[7] Bersanelli M., Travaglino E., Meggendorfer M., Matteuzzi T., Sala C., Mosca E., Chiereghin C., Di Nanni N., Gnocchi M., Zampini M. (2021). Classification and Personalized Prognostic Assessment on the Basis of Clinical and Genomic Features in Myelodysplastic Syndromes. J. Clin. Oncol..

[8] Pellagatti A., Armstrong R.N., Steeples V., Sharma E., Repapi E., Singh S., Sanchi A., Radujkovic A., Horn P., Dolatshad H. (2018). Impact of Spliceosome Mutations on RNA Splicing in Myelodysplasia: Dysregulated Genes/Pathways and Clinical Associations. Blood.

[9] Haferlach T., Nagata Y., Grossmann V., Okuno Y., Bacher U., Nagae G., Schnittger S., Sanada M., Kon A., Alpermann T. (2014). Landscape of Genetic Lesions in 944 Patients with Myelodysplastic Syndromes. Leukemia.

[10] Itzykson R., Kosmider O., Cluzeau T., Mansat-De Mas V., Dreyfus F., Beyne-Rauzy O., Quesnel B., Vey N., Gelsi-Boyer V., Raynaud S. (2011). Impact of TET2 Mutations on Response Rate to Azacitidine in Myelodysplastic Syndromes and Low Blast Count Acute Myeloid Leukemias. Leukemia.

[11] Malcovati L., Stevenson K., Papaemmanuil E., Neuberg D., Bejar R., Boultwood J., Bowen D.T., Campbell P.J., Ebert B.L., Fenaux P. (2020). SF3B1-Mutant MDS as a Distinct Disease Subtype: A Proposal from the International Working Group for the Prognosis of MDS. Blood.

[12] Sallman D.A., List A. (2019). The Central Role of Inflammatory Signaling in the Pathogenesis of Myelodysplastic Syndromes. Blood.

[13] Comont T., Treiner E., Vergez F. (2021). From Immune Dysregulations to Therapeutic Perspectives in Myelodysplastic Syndromes: A Review. Diagnostics.

[14] Zeidan A.M., Shallis R.M., Wang R., Davidoff A., Ma X. Epidemiology of Myelodysplastic Syndromes: Why Characterizing the Beast Is a Prerequisite to Taming It. https://pubmed-ncbi-nlm-nih-gov.proxy.insermbiblio.inist.fr/30314642/.

[15] Chen Y.J., Liao Y.J., Tram V.T.N., Lin C.H., Liao K.C., Liu C.L. (2020). Alterations of Specific Lymphocytic Subsets with Aging and Age-Related Metabolic and Cardiovascular Diseases. Life.

[16] Kovtonyuk L.V., Fritsch K., Feng X., Manz M.G., Takizawa H. (2016). Inflamm-Aging of Hematopoiesis, Hematopoietic Stem Cells, and the Bone Marrow Microenvironment. Front. Immunol..

[17] Fulop T., Larbi A., Dupuis G., Le Page A., Frost E.H., Cohen A.A., Witkowski J.M., Franceschi C. (2018). Immunosenescence and Inflamm-Aging as Two Sides of the Same Coin: Friends or Foes?. Front. Immunol..

[18] Bick A.G., Weinstock J.S., Nandakumar S.K., Fulco C.P., Bao E.L., Zekavat S.M., Szeto M.D., Liao X., Leventhal M.J., Nasser J. (2020). Inherited Causes of Clonal Hematopoiesis in 97,691 TOPMed Whole Genomes. Nature.

[19] Genovese G., Kähler A.K., Handsaker R.E., Lindberg J., Rose S.A., Bakhoum S.F., Chambert K., Mick E., Neale B.M., Fromer M. (2014). Clonal Hematopoiesis and Blood-Cancer Risk Inferred from Blood DNA Sequence. N. Engl. J. Med..

[20] Jaiswal S., Fontanillas P., Flannick J., Manning A., Grauman P.V., Mar B.G., Lindsley R.C., Mermel C.H., Burtt N., Chavez A. (2014). Age-Related Clonal Hematopoiesis Associated with Adverse Outcomes. N. Engl. J. Med..

[21] Jaiswal S., Natarajan P., Silver A.J., Gibson C.J., Bick A.G., Shvartz E., McConkey M., Gupta N., Gabriel S., Ardissino D. Clonal Hematopoiesis and Risk of Atherosclerotic Cardiovascular Disease. https://www-nejm-org.proxy.insermbiblio.inist.fr/doi/10.1056/NEJMoa1701719.

[22] Buscarlet M., Provost S., Zada Y.F., Barhdadi A., Bourgoin V., Lépine G., Mollica L., Szuber N., Dubé M.-P., Busque L. (2017). DNMT3A and TET2 Dominate Clonal Hematopoiesis and Demonstrate Benign Phenotypes and Different Genetic Predispositions. Blood.

[23] Miller P.G., Qiao D., Rojas-Quintero J., Honigberg M.C., Sperling A.S., Gibson C.J., Bick A.G., Niroula A., McConkey M.E., Sandoval B. (2022). Association of Clonal Hematopoiesis with Chronic Obstructive Pulmonary Disease. Blood.

[24] Bonnefond A., Skrobek B., Lobbens S., Eury E., Thuillier D., Cauchi S., Lantieri O., Balkau B., Riboli E., Marre M. (2013). Association between Large Detectable Clonal Mosaicism and Type 2 Diabetes with Vascular Complications. Nat. Genet..

[25] Fuster J.J., Zuriaga M.A., Zorita V., MacLauchlan S., Polackal M.N., Viana-Huete V., Ferrer-Pérez A., Matesanz N., Herrero-Cervera A., Sano S. (2020). TET2-Loss-of-Function-Driven Clonal Hematopoiesis Exacerbates Experimental Insulin Resistance in Aging and Obesity. Cell Rep..

[26] Arends C.M., Galan-Sousa J., Hoyer K., Chan W., Jäger M., Yoshida K., Seemann R., Noerenberg D., Waldhueter N., Fleischer-Notter H. (2018). Hematopoietic Lineage Distribution and Evolutionary Dynamics of Clonal Hematopoiesis. Leukemia.

[27] Buscarlet M., Provost S., Zada Y.F., Bourgoin V., Mollica L., Dubé M.-P., Busque L. (2018). Lineage Restriction Analyses in CHIP Indicate Myeloid Bias for TET2 and Multipotent Stem Cell Origin for DNMT3A. Blood.

[28] Couronné L., Bastard C., Bernard O.A. (2012). TET2 and DNMT3A Mutations in Human T-Cell Lymphoma. N. Engl. J. Med..

[29] Fukumoto K., Nguyen T.B., Chiba S., Sakata-Yanagimoto M. (2018). Review of the Biologic and Clinical Significance of Genetic Mutations in Angioimmunoblastic T-Cell Lymphoma. Cancer Sci..

[30] Gibson C.J., Kim H.T., Zhao L., Murdock H.M., Hambley B., Ogata A., Madero-Marroquin R., Wang S., Green L., Fleharty M. (2022). Donor Clonal Hematopoiesis and Recipient Outcomes After Transplantation. J. Clin. Oncol..

[31] Frick M., Chan W., Arends C.M., Hablesreiter R., Halik A., Heuser M., Michonneau D., Blau O., Hoyer K., Christen F. (2019). Role of Donor Clonal Hematopoiesis in Allogeneic Hematopoietic Stem-Cell Transplantation. J. Clin. Oncol..

[32] Zhang Q., Zhao K., Shen Q., Han Y., Gu Y., Li X., Zhao D., Liu Y., Wang C., Zhang X. (2015). Tet2 Is Required to Resolve Inflammation by Recruiting Hdac2 to Specifically Repress IL-6. Nature.

[33] Cull A.H., Snetsinger B., Buckstein R., Wells R.A., Rauh M.J. (2017). Tet2 Restrains Inflammatory Gene Expression in Macrophages. Exp. Hematol..

[34] Fuster J.J., MacLauchlan S., Zuriaga M.A., Polackal M.N., Ostriker A.C., Chakraborty R., Wu C.-L., Sano S., Muralidharan S., Rius C. (2017). Clonal Hematopoiesis Associated with TET2 Deficiency Accelerates Atherosclerosis Development in Mice. Science.

[35] Sano S., Oshima K., Wang Y., Katanasaka Y., Sano M., Walsh K. (2018). CRISPR-Mediated Gene Editing to Assess the Roles of Tet2 and Dnmt3a in Clonal Hematopoiesis and Cardiovascular Disease. Circ. Res..

[36] Leoni C., Montagner S., Rinaldi A., Bertoni F., Polletti S., Balestrieri C., Monticelli S. (2017). Dnmt3a Restrains Mast Cell Inflammatory Responses. Proc. Natl. Acad. Sci. USA.

[37] Cook E.K., Izukawa T., Young S., Rosen G., Jamali M., Zhang L., Johnson D., Bain E., Hilland J., Ferrone C.K. (2019). Comorbid and Inflammatory Characteristics of Genetic Subtypes of Clonal Hematopoiesis. Blood Adv..

[38] Shi X., Zheng Y., Xu L., Cao C., Dong B., Chen X. (2019). The Inflammatory Cytokine Profile of Myelodysplastic Syndromes. Medicine.

[39] Zhang Z., Li X., Guo J., Xu F., He Q., Zhao Y., Yang Y., Gu S., Zhang Y., Wu L. (2013). Interleukin-17 Enhances the Production of Interferon-γ and Tumour Necrosis Factor-α by Bone Marrow T Lymphocytes from Patients with Lower Risk Myelodysplastic Syndromes. Eur. J. Haematol..

[40] Iwasaki A., Medzhitov R. (2015). Control of Adaptive Immunity by the Innate Immune System. Nat. Immunol..

[41] Nagai Y., Garrett K.P., Ohta S., Bahrun U., Kouro T., Akira S., Takatsu K., Kincade P.W. (2006). Toll-Like Receptors on Hematopoietic Progenitor Cells Stimulate Innate Immune System Replenishment. Immunity.

[42] Esplin B.L., Shimazu T., Welner R.S., Garrett K.P., Nie L., Zhang Q., Humphrey M.B., Yang Q., Borghesi L.A., Kincade P.W. (2011). Chronic Exposure to a TLR Ligand Injures Hematopoietic Stem Cells. J. Immunol..

[43] Luo H., Mu W.-C., Karki R., Chiang H.-H., Mohrin M., Shin J.J., Ohkubo R., Ito K., Kanneganti T.-D., Chen D. (2019). Mitochondrial Stress-Initiated Aberrant Activation of the NLRP3 Inflammasome Regulates the Functional Deterioration of Hematopoietic Stem Cell Aging. Cell Rep..

[44] Barreyro L., Chlon T.M., Starczynowski D.T. (2018). Chronic Immune Response Dysregulation in MDS Pathogenesis. Blood.

[45] Pellagatti A., Cazzola M., Giagounidis A., Perry J., Malcovati L., Della Porta M.G., Jädersten M., Killick S., Verma A., Hellström-Lindberg E. (2010). Deregulated Gene Expression Pathways in Myelodysplastic Syndrome Hematopoietic Stem Cells. Leukemia.

[46] Lc P., Da M., Zj G., Dac F., Mj W., St O., Lg S. Toll-like Receptor and Cytokine Expression throughout the Bone Marrow Differs between Patients with Low- and High-Risk Myelodysplastic Syndromes. https://pubmed-ncbi-nlm-nih-gov.proxy.insermbiblio.inist.fr/35367529/.

[47] Wei Y., Dimicoli S., Bueso-Ramos C., Chen R., Yang H., Neuberg D., Pierce S., Jia Y., Zheng H., Wang H. (2013). Toll-like Receptor Alterations in Myelodysplastic Syndrome. Leukemia.

[48] Monlish D.A., Greenberg Z.J., Bhatt S.T., Leonard K.M., Romine M.P., Dong Q., Bendesky L., Duncavage E.J., Magee J.A., Schuettpelz L.G. (2020). TLR2/6 Signaling Promotes the Expansion of Premalignant Hematopoietic Stem and Progenitor Cells in the NUP98-HOXD13 Mouse Model of MDS. Exp. Hematol..

[49] Monlish D.A., Bhatt S.T., Duncavage E.J., Greenberg Z.J., Keller J.L., Romine M.P., Yang W., Aplan P.D., Walter M.J., Schuettpelz L.G. (2018). Loss of Toll-like Receptor 2 Results in Accelerated Leukemogenesis in the NUP98-HOXD13 Mouse Model of MDS. Blood.

[50] Maratheftis C.I., Andreakos E., Moutsopoulos H.M., Voulgarelis M. (2007). Toll-like Receptor-4 Is Up-Regulated in Hematopoietic Progenitor Cells and Contributes to Increased Apoptosis in Myelodysplastic Syndromes. Clin. Cancer Res..

[51] Ratajczak M.Z., Bujko K., Cymer M., Thapa A., Adamiak M., Ratajczak J., Abdel-Latif A.K., Kucia M. (2020). The Nlrp3 Inflammasome as a “Rising Star” in Studies of Normal and Malignant Hematopoiesis. Leukemia.

[52] Basiorka A.A., McGraw K.L., Eksioglu E.A., Chen X., Johnson J., Zhang L., Zhang Q., Irvine B.A., Cluzeau T., Sallman D.A. (2016). The NLRP3 Inflammasome Functions as a Driver of the Myelodysplastic Syndrome Phenotype. Blood.

[53] Sallman D.A., Cluzeau T., Basiorka A.A., List A. (2016). Unraveling the Pathogenesis of MDS: The NLRP3 Inflammasome and Pyroptosis Drive the MDS Phenotype. Front. Oncol..

[54] Cluzeau T., McGraw K.L., Irvine B., Masala E., Ades L., Basiorka A.A., Maciejewski J., Auberger P., Wei S., Fenaux P. (2017). Pro-Inflammatory Proteins S100A9 and Tumor Necrosis Factor-α Suppress Erythropoietin Elaboration in Myelodysplastic Syndromes. Haematologica.

[55] Giudice V., Wu Z., Kajigaya S., del Pilar Fernandez Ibanez M., Rios O., Cheung F., Ito S., Young N.S. (2019). Circulating S100A8 and S100A9 Protein Levels in Plasma of Patients with Acquired Aplastic Anemia and Myelodysplastic Syndromes. Cytokine.

[56] Velegraki M., Papakonstanti E., Mavroudi I., Psyllaki M., Tsatsanis C., Oulas A., Iliopoulos I., Katonis P., Papadaki H.A. (2013). Impaired Clearance of Apoptotic Cells Leads to HMGB1 Release in the Bone Marrow of Patients with Myelodysplastic Syndromes and Induces TLR4-Mediated Cytokine Production. Haematologica.

[57] Basiorka A.A., McGraw K.L., Abbas-Aghababazadeh F., McLemore A.F., Vincelette N.D., Ward G.A., Eksioglu E.A., Sallman D.A., Al Ali N., Padron E. (2018). Assessment of ASC Specks as a Putative Biomarker of Pyroptosis in Myelodysplastic Syndromes: An Observational Cohort Study. Lancet Haematol..

[58] Chen X., Eksioglu E.A., Zhou J., Zhang L., Djeu J., Fortenbery N., Epling-Burnette P., Van Bijnen S., Dolstra H., Cannon J. (2013). Induction of Myelodysplasia by Myeloid-Derived Suppressor Cells. J. Clin. Invest..

[59] Muto T., Walker C.S., Choi K., Hueneman K., Smith M.A., Gul Z., Garica-Manero G., Ma A., Zheng Y., Statczynowski D.T. (2020). Adaptive Response to Inflammation Contributes to Sustained Myelopoiesis and Confers a Competitive Advantage in Myelodysplastic Syndrome HSCs. Nat. Immunol..

[60] Varney M.E., Niederkorn M., Konno H., Matsumura T., Gohda J., Yoshida N., Akiyama T., Christie S., Fang J., Miller D. (2015). Loss of Tifab, a Del(5q) MDS Gene, Alters Hematopoiesis through Derepression of Toll-like Receptor–TRAF6 Signaling. J. Exp. Med..

[61] Starczynowski D.T., Kuchenbauer F., Argiropoulos B., Sung S., Morin R., Muranyi A., Hirst M., Hogge D., Marra M., Wells R.A. (2010). Identification of MiR-145 and MiR-146a as Mediators of the 5q– Syndrome Phenotype. Nat. Med..

[62] Ribezzo F., Snoeren I.A.M., Ziegler S., Stoelben J., Olofsen P.A., Henic A., Ferreira M.V., Chen S., Stalmann U.S.A., Buesche G. (2019). Rps14, Csnk1a1 and MiRNA145/MiRNA146a Deficiency Cooperate in the Clinical Phenotype and Activation of the Innate Immune System in the 5q- Syndrome. Leukemia.

[63] Mei Y., Zhao B., Basiorka A.A., Yang J., Cao L., Zhang J., List A., Ji P. (2017). Age-Related Inflammatory Bone Marrow Microenvironment Induces Ineffective Erythropoiesis Mimicking Del(5q) MDS. Leukemia.

[64] Keerthivasan G., Mei Y., Zhao B., Zhang L., Harris C.E., Gao J., Basiorka A.A., Schipma M.J., McElherne J., Yang J. (2014). Aberrant Overexpression of CD14 on Granulocytes Sensitizes the Innate Immune Response in MDia1 Heterozygous Del(5q) MDS. Blood.

[65] Mei Y., Basiorka A., Zhao B., Yang J., List A.F., Ji P. (2016). Dual Deficiency of MDia1 and Mir-146a in an Age-Related Inflammatory Bone Marrow Microenvironment Induces Ineffective Erythropoiesis in Del(5q) MDS. Blood.

[66] Schneider R.K., Schenone M., Ferreira M.V., Kramann R., Joyce C.E., Hartigan C., Beier F., Brümmendorf T.H., Germing U., Platzbecker U. (2016). Rps14 Haploinsufficiency Causes a Block in Erythroid Differentiation Mediated by S100A8 and S100A9. Nat. Med..

[67] Stoner S.A., Yan M., Liu K.T.H., Arimoto K.-I., Shima T., Wang H.-Y., Johnson D.T., Bejar R., Jamieson C., Guan K.-L. (2019). Hippo Kinase Loss Contributes to Del(20q) Hematologic Malignancies through Chronic Innate Immune Activation. Blood.

[68] Smith M.A., Choudhary G.S., Pellagatti A., Choi K., Bolanos L.C., Bhagat T.D., Gordon-Mitchell S., Von Ahrens D., Pradhan K., Steeples V. (2019). U2AF1 Mutations Induce Oncogenic IRAK4 Isoforms and Activate Innate Immune Pathways in Myeloid Malignancies. Nat. Cell Biol..

[69] Pollyea D.A., Harris C., Rabe J.L., Hedin B.R., Arras L.D., Katz S., Wheeler E., Bejar R., Walter M.J., Jordan C.T. (2019). Myelodysplastic Syndrome-Associated Spliceosome Gene Mutations Enhance Innate Immune Signaling. Haematologica.

[70] Komrokji R.S., Kulasekararaj A., Al Ali N.H., Kordasti S., Bart-Smith E., Craig B.M., Padron E., Zhang L., Lancet J.E., Pinilla-Ibarz J. (2016). Autoimmune Diseases and Myelodysplastic Syndromes. Am. J. Hematol..

[71] Mekinian A., Grignano E., Braun T., Decaux O., Liozon E., Costedoat-Chalumeau N., Kahn J.-E., Hamidou M., Park S., Puéchal X. (2016). Systemic Inflammatory and Autoimmune Manifestations Associated with Myelodysplastic Syndromes and Chronic Myelomonocytic Leukaemia: A French Multicentre Retrospective Study. Rheumatology.

[72] Zhao L.-P., Boy M., Azoulay C., Clappier E., Sébert M., Amable L., Klibi J., Benlagha K., Espéli M., Balabanian K. (2021). Genomic Landscape of MDS/CMML Associated with Systemic Inflammatory and Autoimmune Disease. Leukemia.

[73] Arinobu Y., Kashiwado Y., Miyawaki K., Ayano M., Kimoto Y., Mitoma H., Akahoshi M., Miyamoto T., Horiuchi T., Akashi K. (2021). Autoimmune Manifestations Associated with Myelodysplastic Syndrome Predict a Poor Prognosis. Medicine.

[74] Giannouli S., Voulgarelis M., Zintzaras E., Tzioufas A.G., Moutsopoulos H.M. (2004). Autoimmune Phenomena in Myelodysplastic Syndromes: A 4-Yr Prospective Study. Rheumatology.

[75] Lietzen L.W., Cronin-Fenton D., Christiansen P., Sørensen H.T., Lash T.L. (2015). Autoimmune Diseases and Breast Cancer Recurrence: A Danish Nationwide Cohort Study. Breast Cancer Res. Treat..

[76] Anderson L.A., Gadalla S., Morton L.M., Landgren O., Pfeiffer R., Warren J.L., Berndt S.I., Ricker W., Parsons R., Engels E.A. (2009). Population-Based Study of Autoimmune Conditions and the Risk of Specific Lymphoid Malignancies. Int. J. Cancer.

[77] Eaton W.W., Rose N.R., Kalaydjian A., Pedersen M.G., Mortensen P.B. (2007). Epidemiology of Autoimmune Diseases in Denmark. J. Autoimmun..

[78] Kristinsson S.Y., Björkholm M., Hultcrantz M., Derolf Å.R., Landgren O., Goldin L.R. (2011). Chronic Immune Stimulation Might Act as a Trigger for the Development of Acute Myeloid Leukemia or Myelodysplastic Syndromes. J. Clin. Oncol..

[79] Anderson L.A., Pfeiffer R.M., Landgren O., Gadalla S., Berndt S.I., Engels E.A. (2009). Risks of Myeloid Malignancies in Patients with Autoimmune Conditions. Br. J. Cancer.

[80] Stahl M., DeVeaux M., de Witte T., Neukirchen J., Sekeres M.A., Brunner A.M., Roboz G.J., Steensma D.P., Bhatt V.R., Platzbecker U. (2018). The Use of Immunosuppressive Therapy in MDS: Clinical Outcomes and Their Predictors in a Large International Patient Cohort. Blood Adv..

[81] Passweg J.R., Giagounidis A.A.N., Simcock M., Aul C., Dobbelstein C., Stadler M., Ossenkoppele G., Hofmann W.-K., Schilling K., Tichelli A. (2010). Immunosuppressive Therapy for Patients with Myelodysplastic Syndrome: A Prospective Randomized Multicenter Phase III Trial Comparing Antithymocyte Globulin Plus Cyclosporine With Best Supportive Care—SAKK 33/99. J. Clin. Oncol..

[82] Komrokji R.S., Mailloux A.W., Chen D.-T., Sekeres M.A., Paquette R., Fulp W.J., Sugimori C., Paleveda-Pena J., Maciejewski J.P., List A.F. (2014). A Phase II Multicenter Rabbit Anti-Thymocyte Globulin Trial in Patients with Myelodysplastic Syndromes Identifying a Novel Model for Response Prediction. Haematologica.

[83] Lim Z.Y., Killick S., Germing U., Cavenagh J., Culligan D., Bacigalupo A., Marsh J., Mufti G.J. (2007). Low IPSS Score and Bone Marrow Hypocellularity in MDS Patients Predict Hematological Responses to Antithymocyte Globulin. Leukemia.

[84] Georgin-Lavialle S., Terrier B., Guedon A.F., Heiblig M., Comont T., Lazaro E., Lacombe V., Terriou L., Ardois S., Bouaziz J.-D. (2022). Further Characterization of Clinical and Laboratory Features in VEXAS Syndrome: Large-Scale Analysis of a Multicentre Case Series of 116 French Patients. Br. J. Dermatol..

[85] Beck D.B., Ferrada M.A., Sikora K.A., Ombrello A.K., Collins J.C., Pei W., Balanda N., Ross D.L., Cardona D.O., Wu Z. (2020). Somatic Mutations in UBA1 and Severe Adult-Onset Autoinflammatory Disease. N. Engl. J. Med..

[86] Zhao L.-P., Schell B., Sébert M., Kim R., Lemaire P., Boy M., Mathis S., Larcher L., Chauvel C., Dhouaieb M.B. (2021). Prevalence of UBA1 Mutations in MDS/CMML Patients with Systemic Inflammatory and Auto-Immune Disease. Leukemia.

[87] Fujii S., Shimizu K., Klimek V., Geller M.D., Nimer S.D., Dhodapkar M.V. (2003). Severe and Selective Deficiency of Interferon-γ-Producing Invariant Natural Killer T Cells in Patients with Myelodysplastic Syndromes. Br. J. Haematol..

[88] Kiladjian J.-J., Bourgeois E., Lobe I., Braun T., Visentin G., Bourhis J.-H., Fenaux P., Chouaib S., Caignard A. (2006). Cytolytic Function and Survival of Natural Killer Cells Are Severely Altered in Myelodysplastic Syndromes. Leukemia.

[89] Hejazi M., Manser A.R., Fröbel J., Kündgen A., Zhao X., Schönberg K., Germing U., Haas R., Gattermann N., Uhrberg M. (2015). Impaired Cytotoxicity Associated with Defective Natural Killer Cell Differentiation in Myelodysplastic Syndromes. Haematologica.

[90] Aggarwal N., Swerdlow S.H., TenEyck S.P., Boyiadzis M., Felgar R.E. (2016). Natural Killer Cell (NK) Subsets and NK-like T-Cell Populations in Acute Myeloid Leukemias and Myelodysplastic Syndromes. Cytometry B Clin. Cytom..

[91] Cianga V.A., Campos Catafal L., Cianga P., Pavel Tanasa M., Cherry M., Collet P., Tavernier E., Guyotat D., Rusu C., Aanei C.M. (2021). Natural Killer Cell Subpopulations and Inhibitory Receptor Dynamics in Myelodysplastic Syndromes and Acute Myeloid Leukemia. Front. Immunol..

[92] Epling-Burnette P.K., Bai F., Painter J.S., Rollison D.E., Salih H.R., Krusch M., Zou J., Ku E., Zhong B., Boulware D. (2007). Reduced Natural Killer (NK) Function Associated with High-Risk Myelodysplastic Syndrome (MDS) and Reduced Expression of Activating NK Receptors. Blood.

[93] Carlsten M., Järås M. (2019). Natural Killer Cells in Myeloid Malignancies: Immune Surveillance, NK Cell Dysfunction, and Pharmacological Opportunities to Bolster the Endogenous NK Cells. Front. Immunol..

[94] Sohlberg E., Pfefferle A., Andersson S., Baumann B.C., Hellström-Lindberg E., Malmberg K.-J. (2015). Imprint of 5-Azacytidine on the Natural Killer Cell Repertoire during Systemic Treatment for High-Risk Myelodysplastic Syndrome. Oncotarget.

[95] Tsirogianni M., Grigoriou E., Kapsimalli V., Dagla K., Stamouli M., Gkirkas K., Konsta E., Karagiannidou A., Gkodopoulos K., Stavroulaki G. (2019). Natural Killer Cell Cytotoxicity Is a Predictor of Outcome for Patients with High Risk Myelodysplastic Syndrome and Oligoblastic Acute Myeloid Leukemia Treated with Azacytidine. Leuk. Lymphoma.

[96] Farhood B., Najafi M., Mortezaee K. (2019). CD8+ Cytotoxic T Lymphocytes in Cancer Immunotherapy: A Review. J. Cell. Physiol..

[97] Sand K., Theorell J., Bruserud Ø., Bryceson Y.T., Kittang A.O. (2016). Reduced Potency of Cytotoxic T Lymphocytes from Patients with High-Risk Myelodysplastic Syndromes. Cancer Immunol. Immunother..

[98] Tao J., Li L., Wang Y., Fu R., Wang H., Shao Z. (2016). Increased TIM3+CD8+T Cells in Myelodysplastic Syndrome Patients Displayed Less Perforin and Granzyme B Secretion and Higher CD95 Expression. Leuk. Res..

[99] Cheng P., Eksioglu E.A., Chen X., Kandell W., Le Trinh T., Cen L., Qi J., Sallman D.A., Zhang Y., Tu N. (2019). S100A9-Induced Overexpression of PD-1/PD-L1 Contributes to Ineffective Hematopoiesis in Myelodysplastic Syndromes. Leukemia.

[100] Fu R., Li L., Hu J., Wang Y., Tao J., Liu H., Liu Z., Zhang W. (2019). Elevated TIM3 Expression of T Helper Cells Affects Immune System in Patients with Myelodysplastic Syndrome. J. Investig. Med..

[101] Tao J., Han D., Gao S., Zhang W., Yu H., Liu P., Fu R., Li L., Shao Z. (2020). CD8+ T Cells Exhaustion Induced by Myeloid-derived Suppressor Cells in Myelodysplastic Syndromes Patients Might Be through TIM3/Gal-9 Pathway. J. Cell. Mol. Med..

[102] Meng F., Li L., Lu F., Yue J., Liu Z., Zhang W., Fu R. (2020). Overexpression of TIGIT in NK and T Cells Contributes to Tumor Immune Escape in Myelodysplastic Syndromes. Front. Oncol..

[103] Zhang Z., Liu S., Zhang B., Qiao L., Zhang Y., Zhang Y. (2020). T Cell Dysfunction and Exhaustion in Cancer. Front. Cell Dev. Biol..

[104] Yang X., Ma L., Zhang X., Huang L., Wei J. (2022). Targeting PD-1/PD-L1 Pathway in Myelodysplastic Syndromes and Acute Myeloid Leukemia. Exp. Hematol. Oncol..

[105] Giovazzino A., Leone S., Rubino V., Palatucci A.T., Cerciello G., Alfinito F., Pane F., Ruggiero G., Terrazzano G. (2018). Reduced Regulatory T Cells (Treg) in Bone Marrow Preferentially Associate with the Expansion of Cytotoxic T Lymphocytes in Low Risk MDS Patients. Br. J. Haematol..

[106] Fozza C., Contini S., Galleu A., Pina Simula M., Virdis P., Bonfigli S., Longinotti M. (2009). Patients with Myelodysplastic Syndromes Display Several T-Cell Expansions, Which Are Mostly Polyclonal in the CD4+ Subset and Oligoclonal in the CD8+ Subset. Exp. Hematol..

[107] Sloand E.M., Melenhorst J.J., Tucker Z.C.G., Pfannes L., Brenchley J.M., Yong A., Visconte V., Wu C., Gostick E., Scheinberg P. (2011). T-Cell Immune Responses to Wilms Tumor 1 Protein in Myelodysplasia Responsive to Immunosuppressive Therapy. Blood.

[108] Suwabe T., Shibasaki Y., Sato H., Tamura S., Katagiri T., Nemoto H., Kasami T., Kozakai T., Nanba A., Kitajima T. (2021). WT1-Specific CD8 + Cytotoxic T Cells with the Capacity for Antigen-Specific Expansion Accumulate in the Bone Marrow in MDS. Int. J. Hematol..

[109] Tanaka T.N., Ferrari V., Tarke A., Fields H., Ferrari L., Ferrari F., McCarthy C.L., Sanchez A.P., Vitiello A., Lane T.A. (2021). Adoptive Transfer of Neoantigen-Specific T-Cell Therapy Is Feasible in Older Patients with Higher-Risk Myelodysplastic Syndrome. Cytotherapy.

[110] Keilholz U., Letsch A., Busse A., Asemissen A.M., Bauer S., Blau I.W., Hofmann W.-K., Uharek L., Thiel E., Scheibenbogen C. (2009). A Clinical and Immunologic Phase 2 Trial of Wilms Tumor Gene Product 1 (WT1) Peptide Vaccination in Patients with AML and MDS. Blood.

[111] Griffiths E.A., Srivastava P., Matsuzaki J., Brumberger Z., Wang E.S., Kocent J., Miller A., Roloff G.W., Wong H.Y., Paluch B.E. (2018). NY-ESO-1 Vaccination in Combination with Decitabine Induces Antigen-Specific T-Lymphocyte Responses in Patients with Myelodysplastic Syndrome. Clin. Cancer Res..

[112] Ferrari V., Tarke A., Fields H., Ferrari L., Conley T., Ferrari F., Koşaloğlu-Yalçın Z., Sette A., Peters B., McCarthy C.L. (2021). In Vitro Induction of Neoantigen-Specific T Cells in Myelodysplastic Syndrome, a Disease with Low Mutational Burden. Cytotherapy.

[113] Li T., Wu B., Yang T., Zhang L., Jin K. (2020). The Outstanding Antitumor Capacity of CD4+ T Helper Lymphocytes. Biochim. Biophys. Acta BBA—Rev. Cancer.

[114] Wang X., Wu D.P., He G., Miao M., Sun A. (2005). Research of Subset and Function of Th Cells in Bone Marrow of Myelodysplastic Syndrome Patients. Blood.

[115] Li J., Yue L., Wang H., Liu C., Liu H., Tao J., Qi W., Wang Y., Zhang W., Fu R. (2016). Th17 Cells Exhibit Antitumor Effects in MDS Possibly through Augmenting Functions of CD8+ T Cells. J. Immunol. Res..

[116] Shao L., Zhang L., Hou Y., Yu S., Liu X., Huang X., Sun Y., Tian T., He N., Ma D. (2012). Th22 Cells as Well as Th17 Cells Expand Differentially in Patients with Early-Stage and Late-Stage Myelodysplastic Syndrome. PLoS ONE.

[117] Kordasti S.Y., Ingram W., Hayden J., Darling D., Barber L., Afzali B., Lombardi G., Wlodarski M.W., Maciejewski J.P., Farzaneh F. (2007). CD4+CD25high Foxp3+ Regulatory T Cells in Myelodysplastic Syndrome (MDS). Blood.

[118] Kotsianidis I., Bouchliou I., Nakou E., Spanoudakis E., Margaritis D., Christophoridou A.V., Anastasiades A., Tsigalou C., Bourikas G., Karadimitris A. (2009). Kinetics, Function and Bone Marrow Trafficking of CD4+CD25+FOXP3+ Regulatory T Cells in Myelodysplastic Syndromes (MDS). Leukemia.

[119] Bouchliou I., Miltiades P., Nakou E., Spanoudakis E., Goutzouvelidis A., Vakalopoulou S., Garypidou V., Kotoula V., Bourikas G., Tsatalas C. (2011). Th17 and Foxp3+ T Regulatory Cell Dynamics and Distribution in Myelodysplastic Syndromes. Clin. Immunol..

[120] Mailloux A.W., Sugimori C., Komrokji R.S., Yang L., Maciejewski J.P., Sekeres M.A., Paquette R., Loughran T.P., List A.F., Epling-Burnette P.K. (2012). Expansion of Effector Memory Regulatory T Cells Represents a Novel Prognostic Factor in Lower Risk Myelodysplastic Syndrome. J. Immunol..

[121] Kahn J.D., Chamuleau M.E.D., Westers T.M., Van de Ven P.M., van Dreunen L., van Spronsen M., Ossenkoppele G.J., van de Loosdrecht A.A. (2015). Regulatory T Cells and Progenitor B Cells Are Independent Prognostic Predictors in Lower Risk Myelodysplastic Syndromes. Haematologica.

[122] Sallman D.A., McLemore A.F., Aldrich A.L., Komrokji R.S., McGraw K.L., Dhawan A., Geyer S., Hou H.-A., Eksioglu E.A., Sullivan A. (2020). TP53 Mutations in Myelodysplastic Syndromes and Secondary AML Confer an Immunosuppressive Phenotype. Blood.

[123] Mailloux A.W., Epling-Burnette P.K. (2013). Effector Memory Regulatory T-Cell Expansion Marks a Pivotal Point of Immune Escape in Myelodysplastic Syndromes. OncoImmunology.

[124] Costantini B., Kordasti S.Y., Kulasekararaj A.G., Jiang J., Seidl T., Abellan P.P., Mohamedali A., Thomas N.S.B., Farzaneh F., Mufti G.J. (2013). The Effects of 5-Azacytidine on the Function and Number of Regulatory T Cells and T-Effectors in Myelodysplastic Syndrome. Haematologica.

[125] Bontkes H.J., Ruben J.M., Alhan C., Westers T.M., Ossenkoppele G.J., van de Loosdrecht A.A. (2012). Azacitidine Differentially Affects CD4pos T-Cell Polarization in Vitro and in Vivo in High Risk Myelodysplastic Syndromes. Leuk. Res..

[126] Veglia F., Sanseviero E., Gabrilovich D.I. (2021). Myeloid-Derived Suppressor Cells in the Era of Increasing Myeloid Cell Diversity. Nat. Rev. Immunol..

[127] Barreau S., Green A.S., Dussiau C., Alary A.-S., Raimbault A., Mathis S., Willems L., Bouscary D., Kosmider O., Bardet V. (2020). Phenotypic Landscape of Granulocytes and Monocytes by Multiparametric Flow Cytometry: A Prospective Study of a 1-Tube Panel Strategy for Diagnosis and Prognosis of Patients with MDS. Cytometry B Clin. Cytom..

[128] Kittang A.O., Kordasti S., Sand K.E., Costantini B., Kramer A.M., Perezabellan P., Seidl T., Rye K.P., Hagen K.M., Kulasekararaj A. (2015). Expansion of Myeloid Derived Suppressor Cells Correlates with Number of T Regulatory Cells and Disease Progression in Myelodysplastic Syndrome. Oncoimmunology.

[129] Qi X., Jiang H., Liu P., Xie N., Fu R., Wang H., Liu C., Zhang T., Wang H., Shao Z. (2020). Increased Myeloid-Derived Suppressor Cells in Patients with Myelodysplastic Syndromes Suppress CD8+ T Lymphocyte Function through the STAT3-ARG1 Pathway. Leuk. Lymphoma.

[130] Kapor S., Santibanez J.F. (2021). Myeloid-Derived Suppressor Cells and Mesenchymal Stem/Stromal Cells in Myeloid Malignancies. J. Clin. Med..

[131] Han D., Tao J., Fu R., Shao Z. (2020). Myeloid-Derived Suppressor Cell Cytokine Secretion as Prognostic Factor in Myelodysplastic Syndromes. Innate Immun..

[132] Gleason M.K., Ross J.A., Warlick E.D., Lund T.C., Verneris M.R., Wiernik A., Spellman S., Haagenson M.D., Lenvik A.J., Litzow M.R. (2014). CD16xCD33 Bispecific Killer Cell Engager (BiKE) Activates NK Cells against Primary MDS and MDSC CD33+ Targets. Blood.

[133] Eksioglu E.A., Chen X., Heider K.-H., Rueter B., McGraw K.L., Basiorka A.A., Wei M., Burnette A., Cheng P., Lancet J. (2017). Novel Therapeutic Approach to Improve Hematopoiesis in Low Risk MDS by Targeting MDSCs with the Fc-Engineered CD33 Antibody BI 836858. Leukemia.

[134] Sarhan D., Brandt L., Felices M., Guldevall K., Lenvik T., Hinderlie P., Curtsinger J., Warlick E., Spellman S.R., Blazar B.R. (2018). 161533 TriKE Stimulates NK-Cell Function to Overcome Myeloid-Derived Suppressor Cells in MDS. Blood Adv..

[135] Cheng P., Chen X., Dalton R., Calescibetta A., So T., Gilvary D., Ward G., Smith V., Eckard S., Foxa J.A. (2022). Immunodepletion of MDSC by AMV564, a Novel Bivalent Bispecific CD33/CD3 T-Cell Engager Ex Vivo in MDS and Melanoma. Mol. Ther. J. Am. Soc. Gene Ther..

[136] Han Q., Sun Z., Liu L., Chen B., Cao Y., Li K., Zhao R.C. Impairment in Immuno-Modulatory Function of Flk1(+)CD31(−)CD34(−) MSCs from MDS-RA Patients. https://pubmed-ncbi-nlm-nih-gov.proxy.insermbiblio.inist.fr/17360037/.

[137] Zambetti N.A., Ping Z., Chen S., Kenswil K.J.G., Mylona M.A., Sanders M.A., Hoogenboezem R.M., Bindels E.M., Adisty M.N., Van Strien P.M.H. (2016). Mesenchymal Inflammation Drives Genotoxic Stress in Hematopoietic Stem Cells and Predicts Disease Evolution in Human Pre-Leukemia. Cell Stem Cell.

[138] Allampallam K., Shetty V., Mundle S., Dutt D., Kravitz H., Reddy P.L., Alvi S., Galili N., Saberwal G.S., Anthwal S. Biological Significance of Proliferation, Apoptosis, Cytokines, and Monocyte/Macrophage Cells in Bone Marrow Biopsies of 145 Patients with Myelodysplastic Syndrome. https://pubmed-ncbi-nlm-nih-gov.proxy.insermbiblio.inist.fr/11999358/.

[139] Han Y., Wang H., Shao Z. (2016). Monocyte-Derived Macrophages Are Impaired in Myelodysplastic Syndrome. J. Immunol. Res..

[140] Zhang G., Yang L., Han Y., Niu H., Yan L., Shao Z., Xing L., Wang H. Abnormal Macrophage Polarization in Patients with Myelodysplastic Syndrome. https://www.hindawi.com/journals/mi/2021/9913382/.

[141] Bento L.C., Bacal N.S., Rocha F.A., Severino P., Marti L.C. (2020). Bone Marrow Monocytes and Derived Dendritic Cells from Myelodysplastic Patients Have Functional Abnormalities Associated with Defective Response to Bacterial Infection. J. Immunol..

[142] Ma L., Ceuppens J., Kasran A., Delforge M., Boogaerts M., Vandenberghe P. Immature and Mature Monocyte-Derived Dendritic Cells in Myelodysplastic Syndromes of Subtypes Refractory Anemia or Refractory Anemia with Ringed Sideroblasts Display an Altered Cytokine Profile. https://pubmed-ncbi-nlm-nih-gov.proxy.insermbiblio.inist.fr/17188353/.

[143] Selimoglu-Buet D., Wagner-Ballon O., Saada V., Bardet V., Itzykson R., Bencheikh L., Morabito M., Met E., Debord C., Benayoun E. (2015). Characteristic Repartition of Monocyte Subsets as a Diagnostic Signature of Chronic Myelomonocytic Leukemia. Blood.

[144] Wagner-Ballon O., Bettelheim P., Lauf J., Bellos F., Della Porta M., Travaglino E., Subira D., Lopez I.N., Tarfi S., Westers T.M. (2021). ELN IMDS Flow Working Group Validation of the Monocyte Assay for Chronic Myelomonocytic Leukemia Diagnosis by Flow Cytometry. Cytometry B Clin. Cytom..

[145] Selimoglu-Buet D., Badaoui B., Benayoun E., Toma A., Fenaux P., Quesnel B., Etienne G., Braun T., Abermil N., Morabito M. (2017). Accumulation of Classical Monocytes Defines a Subgroup of MDS That Frequently Evolves into CMML. Blood.

[146] Velegraki M., Papakonstantinou N., Kalaitzaki L., Ntoufa S., Laidou S., Tsagiopoulou M., Bizymi N., Damianaki A., Mavroudi I., Pontikoglou C. Increased Proportion and Altered Properties of Intermediate Monocytes in the Peripheral Blood of Patients with Lower Risk Myelodysplastic Syndrome. https://pubmed-ncbi-nlm-nih-gov.proxy.insermbiblio.inist.fr/33032166/.

[147] Galon J., Costes A., Sanchez-Cabo F., Kirilovsky A., Mlecnik B., Lagorce-Pagès C., Tosolini M., Camus M., Berger A., Wind P. (2006). Type, Density, and Location of Immune Cells Within Human Colorectal Tumors Predict Clinical Outcome. Science.

[148] Lanzi A., Pagès F., Lagorce-Pagès C., Galon J. (2020). The Consensus Immunoscore: Toward a New Classification of Colorectal Cancer. OncoImmunology.

[149] Mlecnik B., Bifulco C., Bindea G., Marliot F., Lugli A., Lee J.J., Zlobec I., Rau T.T., Berger M.D., Nagtegaal I.D. (2020). Multicenter International Society for Immunotherapy of Cancer Study of the Consensus Immunoscore for the Prediction of Survival and Response to Chemotherapy in Stage III Colon Cancer. J. Clin. Oncol..

[150] Yalcin S., Philip P.A., Athanasiadis I., Bazarbashi S., Shamseddine A. (2021). Classification of Early-Stage Colon Cancer with Immunoscore^®^: Clinical Evidence and Case Studies. Future Oncol..

[151] Van de Loosdrecht A.A., Kern W., Porwit A., Valent P., Kordasti S., Cremers E., Alhan C., Duetz C., Dunlop A., Hobo W. (2021). Clinical Application of Flow Cytometry in Patients with Unexplained Cytopenia and Suspected Myelodysplastic Syndrome: A Report of the European LeukemiaNet International MDS-Flow Cytometry Working Group. Cytometry B Clin. Cytom..

[152] Winter S., Shoaie S., Kordasti S., Platzbecker U. (2020). Integrating the “Immunome” in the Stratification of Myelodysplastic Syndromes and Future Clinical Trial Design. J. Clin. Oncol..

[153] Colmenares R., Álvarez N., Barrio S., Martínez-López J., Ayala R. (2022). The Minimal Residual Disease Using Liquid Biopsies in Hematological Malignancies. Cancers.

[154] Westers T.M., Cremers E.M.P., Oelschlaegel U., Johansson U., Bettelheim P., Matarraz S., Orfao A., Moshaver B., Brodersen L.E., Loken M.R. (2017). Immunophenotypic Analysis of Erythroid Dysplasia in Myelodysplastic Syndromes. A Report from the IMDSFlow Working Group. Haematologica.

[155] Bardet V., Wagner-Ballon O., Guy J., Morvan C., Debord C., Trimoreau F., Benayoun E., Chapuis N., Freynet N., Rossi C. (2015). Multicentric Study Underlining the Interest of Adding CD5, CD7 and CD56 Expression Assessment to the Flow Cytometric Ogata Score in Myelodysplastic Syndromes and Myelodysplastic/Myeloproliferative Neoplasms. Haematologica.

[156] Mathis S., Chapuis N., Debord C., Rouquette A., Radford-Weiss I., Park S., Dreyfus F., Lacombe C., Béné M.C., Kosmider O. (2013). Flow Cytometric Detection of Dyserythropoiesis: A Sensitive and Powerful Diagnostic Tool for Myelodysplastic Syndromes. Leukemia.

[157] Shameli A., Dharmani-Khan P., Luider J., Auer I., Shabani-Rad M.-T. (2021). Exploring Blast Composition in Myelodysplastic Syndromes and Myelodysplastic/Myeloproliferative Neoplasms: CD45RA and CD371 Improve Diagnostic Value of Flow Cytometry through Assessment of Myeloblast Heterogeneity and Stem Cell Aberrancy. Cytometry B Clin. Cytom..

[158] Bachas C., Duetz C., van Spronsen M.F., Verhoeff J., Garcia Vallejo J.J., Jansen J.H., Cloos J., Westers T.M., van de Loosdrecht A.A. (2022). Characterization of Myelodysplastic Syndromes Hematopoietic Stem and Progenitor Cells Using Mass Cytometry. Cytometry B Clin. Cytom..

[159] Behbehani G.K., Finck R., Samusik N., Sridhar K., Fantl W.J., Greenberg P.L., Nolan G.P. (2020). Profiling Myelodysplastic Syndromes by Mass Cytometry Demonstrates Abnormal Progenitor Cell Phenotype and Differentiation. Cytometry B Clin. Cytom..

